# Improvement of variant reclassification in genetic neurodevelopmental conditions

**DOI:** 10.1016/j.gimo.2024.101845

**Published:** 2024-04-09

**Authors:** Michelle Kowanda, Rebecca Sheedy Smith, Jamie Lundy, Catherine Kentros, Elisheva Kleinman, Lauren Kasparson Walsh, Gerhard Schratt, Cora M. Taylor, Wendy K. Chung

**Affiliations:** 1 Geisinger Autism & Developmental Medicine Institute, Lewisburg, PA; 2 Laboratory of Systems Neuroscience, Institute for Neuroscience, Department of Health Science and Technology, Swiss Federal Institute of Technology ETH, Zurich, Switzerland; 3 Rare Disease Translational Center, The Jackson Laboratory, Bar Harbor, ME; 4 Department of Pediatrics, Columbia University Irving Medical Center, New York, NY; 5 Department of Pediatrics, Boston Children’s Hospital, Boston, MA; 6 Faculty of Pediatrics, Harvard Medical School, Boston, MA

**Keywords:** Autism, Genetic neurodevelopmental disorders, Variant interpretation, Variant pathogenicity, Variants of uncertain significance

## Abstract

**Purpose:**

Limited knowledge about disease mechanisms, few published cases, and the lack of functional assessment of variants for neurodevelopmental genetic disorders challenge diagnostic classification for variants and increase the frequency of variants of uncertain significance (VUS). Because inheritance patterns aid in variant interpretation for neurodevelopmental conditions, genetic testing including only the proband leads to larger numbers of VUS than testing strategies that include the parents.

**Methods:**

We reinterpreted genetic variants submitted to the Simons Searchlight research registry using American College of Medical Genetics and Genomics variant interpretation guidelines, familial cascade testing, and literature curation with annual VUS reevaluation.

**Results:**

Simons Searchlight has independently evaluated 2834 genetic laboratory reports; 20.4% of variants (1.7% copy-number variants and 18.7% monogenic variants) were reclassified with 230 upgrades and 173 downgrades in pathogenicity. Of 351 monogenic VUS on the original clinical test report, 25.4% were reclassified as likely pathogenic or pathogenic. VUS in *SCN2A*, *SLC6A1*, or *STXBP1* were more likely to have VUS reclassified compared with variants in other genes.

**Conclusion:**

Regular reevaluation of neurodevelopmental genetic variants can be helpful because relevant variant reclassifications occur frequently and may affect clinical care. Simons Searchlight contributes to the international neurodevelopmental community by systematically reviewing uncertain variants annually and providing reclassified variants to participants, researchers, and ClinVar.

## Introduction

The American College of Medical Genetics and Genomics (ACMG) guidelines for variant interpretation provides standards for genetic variant classification. Variant classifications may change over time as new evidence emerges.^1^^,^^2^ The frequency and direction of reclassifying variants varies with the clinical indication/set of genes. It is widely known that 73% to 91%^3^, ^4^, ^5^ of variants are downgraded from variants of uncertain significance (VUS) to “benign” for germline cancer genetic testing, with a median time of reclassification of 1.17 years.^5^ For other clinical indications, variant classification and interpretation have not been as quick to change. For example, arrhythmia and cardiomyopathy variants are highly unlikely to be reclassified within a 3-year period.^6^ Furthermore, 71.2% of genetic variants found in inherited arrhythmias, which were initially classified as pathogenic or likely pathogenic in 2010, were downgraded to VUS in 2020 with updated ACMG guidelines.^7^ Examination of variant reclassification by clinical indication may help explain and set expectations about the limitations of genetic testing to patients, inform result disclosure sessions, and guide variant reanalysis strategies and policies moving forward.

To study the frequency of variant reclassification for neurodevelopmental conditions, we investigated the genetic data available in the international Simons Searchlight research registry. This registry of neurodevelopmental genetic conditions is supported by Simons Foundation Autism Research Initiative.^8^ Data and original variant classifications were obtained from genetic laboratory reports submitted by participants. Variant classifications were independently reviewed by a board-certified genetic counselor. Simons Searchlight staff abstract and verify genetic variants from genetic laboratory reports or detailed clinical notes for neurodevelopmental conditions, providing a unique opportunity to assess variant reinterpretation within a large group of rare diseases with shared features.

## Materials and Methods

### Participants

Simons Searchlight is an online patient registry for individuals with highly penetrant genetic variants contributing to neurodevelopmental phenotypes, formerly known as Simons Variation in Individuals Project.^8^ A list of genetic conditions included in Simons Searchlight can be found at www.sfari.org/resource/simons-searchlight. Participants currently can participate in English, Dutch, French, and Spanish.

After participant registration and consent, externally performed clinical or research genetic test reports were submitted through an online platform. Participants without a copy of their genetic laboratory reports provided digital consent for Simons Searchlight research staff to request records on their behalf; participants were then provided with their original laboratory report from Simons Searchlight research staff once obtained.

### Review of genetic testing reports

Each external genetic test report or detailed clinical note was reviewed by a certified genetic counselor to ensure study eligibility. Genetic test reports submitted in languages other than English were viewed in AdobePro and translated by Google Translate. Participant data were extracted from the report and entered into the Simons Searchlight database, including the date of testing, testing laboratory, testing methodology, all genetic findings, and any evidence applied toward the classification of reported variants. Select clinical notes with thorough variant descriptions were accepted in lieu of a laboratory report. Individuals with pathogenic, likely pathogenic, and VUS in genetic conditions on the Simons Searchlight inclusion list are eligible for participation.

Genetic counselors used publicly available databases, including the Genome Aggregation Database (gnomAD),^9^ ClinVar,^10^ Human Gene Mutation Database (HGMD),^11^ University of California Santa Cruz (UCSC) Genome Browser,^12^ and Clinical Genome Resource (ClinGen),^13^ Alamut Visual Plus version 1.6.1, SOPHiA GENETICS, and internal data to assess variants. Evidence was used to provide an independent variant classification in accordance with current ACMG variant interpretation guidelines.^14^^,^^15^ In addition to preliminary review and independent interpretation, any variants classified as VUS are reviewed on an annual basis. Approved researchers can obtain the Simons Searchlight population data set described in this study by applying at https://base.sfari.org. The policy of the Simons Foundation is to withhold data for genetic conditions with fewer than 5 participants in an effort to protect participant privacy.

### Cascade testing

Targeted genetic testing was offered and paid for by the study for first-degree relatives for copy-number variants (CNVs) that were inherited or for CNVs with unknown inheritance. Targeted variant testing was offered for variants associated with monogenic conditions if: the variant was inherited, was classified as VUS, or would be classified as likely pathogenic if de novo inheritance would upgrade the classification. Testing is also offered to participants with a likely pathogenic variant if de novo inheritance will reclassify a variant to pathogenic. For parents reporting another affected sibling of a proband with a de novo pathogenic variant, participants were suggested to request comprehensive clinical testing from their physician because of the decreased chance of yielding a result with targeted testing. Not all eligible participants opted to pursue cascade testing.

### Analysis

Genetic variants included in Simons Searchlight data release version 11 (V11) were examined. Genetic conditions with fewer than 5 individuals are not included in the data releases to protect participant privacy. All participants with a genetic variant downgraded to likely benign or benign are excluded from the Simons Searchlight data release, and registry participation. Variants submitted without ACMG classification are reviewed and classified; these reports are not counted as “reclassified.” To collect variant classifications from ClinVar, ClinVar Miner was used for data filtering of data sets for ClinVar version December 31, 2022.^16^ Participants are notified of the reclassification by a Simons Searchlight genetic counselor through email in the language used by the research participant. Study staff use Google Translate to translate emails from English to other languages. Participants are instructed to follow up with their provider to seek clinical confirmation of the reclassification. VUS upgraded or downgraded were counted as reclassified in both ClinVar and Simons Searchlight. To examine the significance between VUS reclassification and variants submitted, a χ^2^ test was performed.

## Results

Genetic laboratory reports were submitted from 71 different countries for 91 genetic conditions associated with autism and neurodevelopmental disorders in data release V11 (Supplemental Table 1). The spectrum of neurodevelopmental phenotypes has been described elsewhere, with participants reporting more medical complications on average than the general population, including seizures, intellectual disability, and other comorbidities.^17^

**Figure 1:**
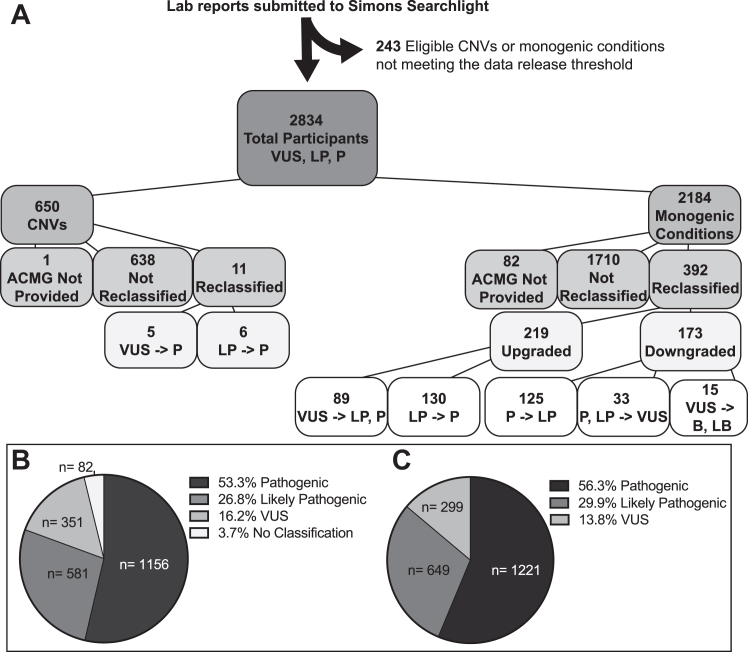
**Simons Searchlight genetic laboratory reports reviewed and the resulting variant classifications upon revision.** A. Genetic laboratory reports are reviewed and curated prior to approving research participation. Genetic conditions with fewer than 5 participants do not meet the data release threshold and are not included in data releases until they accumulate 5 or more participants. B. Breakdown of variant categories before reclassification within version 11 Simons Searchlight data release and (C) post-reclassification categories of current American College of Medical Genetics and Genomics (ACMG) classification. B, benign; CNV, copy-number variant; LB, likely benign; LP, likely pathogenic; P, pathogenic; VUS, variant of uncertain significance.

Between December 2013 and December 2022, 3077 genetic testing reports were submitted to Simons Searchlight for conditions included in our study (Figure 1A). Two hundred forty-three participants were withheld from release until their condition met the minimum threshold of 5 participants.

Recurrent CNVs make up 23.1% of the current data release (650/2819) and are less likely to be reclassified (1.7% [11/650]). Monogenic conditions were more likely to be submitted with no ACMG classification or to be reclassified compared with CNVs. Monogenic conditions were reclassified 18.7% (392/2102) of the time, with more overall upgrades than downgrades. There were 89 clinically significant upgrades of VUS to likely pathogenic or pathogenic. The median time for revision was 2 years (range 0-12 years).

Of the ACMG-classified monogenic variants, 25.4% (89/351) of VUS were upgraded with a median revision time of 3 years, whereas 4.3% (15/351) were downgraded to likely benign or benign. A genetic report without ACMG classification was classified as VUS in 23.2% (19/82) of cases after review and otherwise were pathogenic/likely pathogenic (Figure 1B). The majority (86.2% [1870/2169]) of monogenic variants after review were either pathogenic or likely pathogenic (Figure 1C).

**Figure 2:**
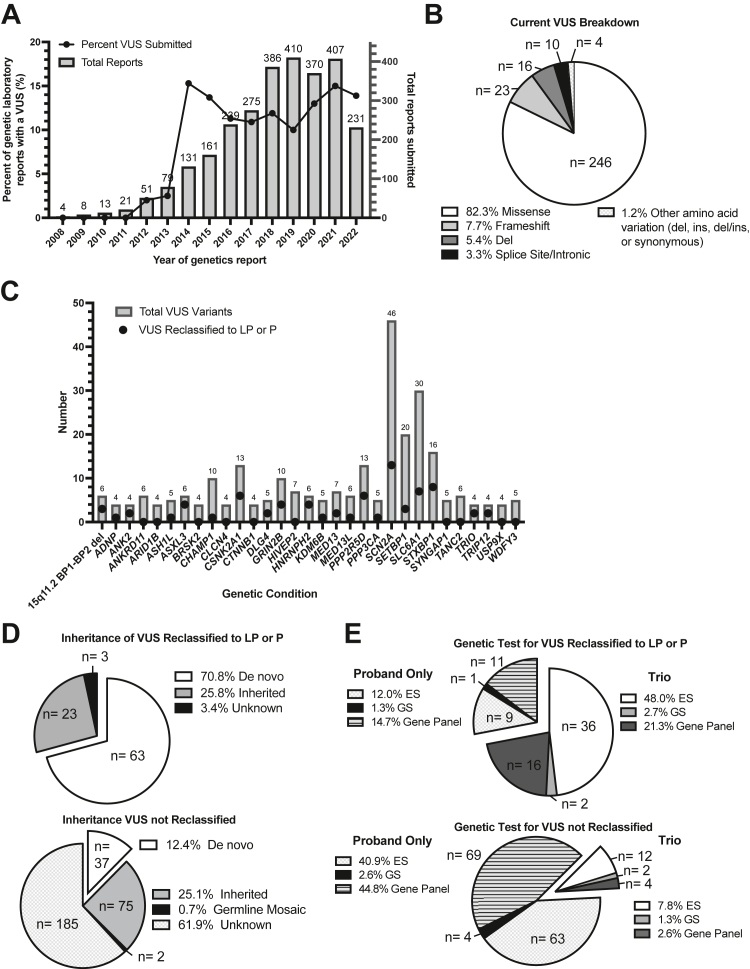
**Analysis of Simons Searchlight VUS variants, pre- and post-variant interpretation.** A. Date (by year only) on the submitted genetics laboratory report (right *y*-axis), and the percent of variants of uncertain significance (VUS) in that year (left *y*-axis). B. Type of variant among VUS (AA, amino acid). C. Only including genetic conditions with >3 VUS submitted to the registry, number of VUS upgraded of the VUS reassessed by gene. D. Inheritance status of VUS that were reclassified (upper panel) and not reclassified (lower panel), and (E) type of genetic testing for all variants that were (upper panel) and were not reclassified (lower panel). del, deletion; ES, exome sequencing; GS, genome sequencing; ins, insertion; LP, likely pathogenic; P, pathogenic.

According to the dates on the genetic testing reports, there was an increase in genetic testing and study entry for Simons Searchlight conditions over time, coinciding with an increase in VUS results returned at the time of testing to affected individuals (Figure 2A). This also corresponds to the expansion of our genetic condition inclusion list in 2014. Thirty-four records did not have a date provided.

After reclassification, the majority of the remaining 299 VUS were missense variants (Figure 2B). *SCN2A* was the genetic condition most frequently associated with VUS classifications, with 46 VUS submitted and 13 upgraded to likely pathogenic or pathogenic (Figure 2C). Notably, 68 of the 91 genetic conditions included in this analysis had at least 1 genetic laboratory report submitted with a VUS (data not shown). The majority (70.8%) of VUS reclassified were de novo variants. For VUS that were not reclassified, most were inheritance unknown 61.9% (Figure 2D) and had exome sequencing (ES) or a gene sequencing panel for the proband only (Figure 2E).

**Figure 3:**
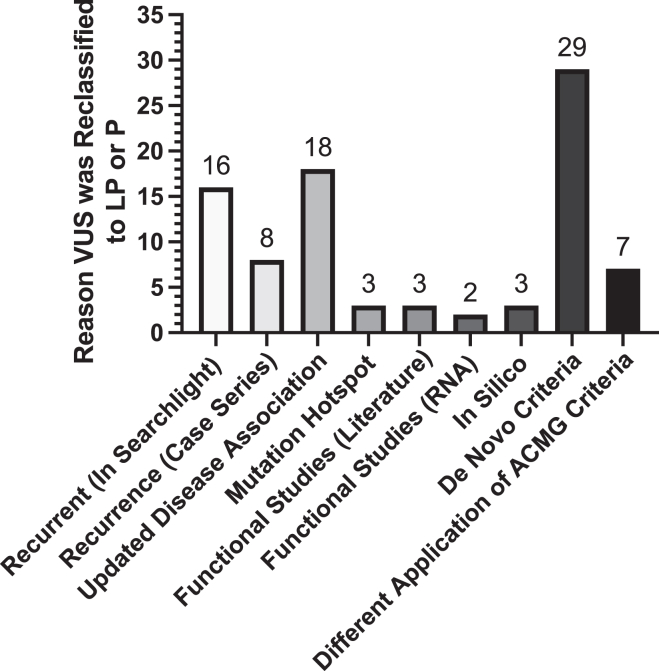
**Criteria applied to aid in VUS reclassification for the 89 variants that were upgraded to likely pathogenic or pathogenic.** LP, likely pathogenic; P, pathogenic; VUS, variants of uncertain significance.

Revisions in classification were driven by published cases or functional testing in the literature, available inheritance information, and variant submissions of recurrent variants within our database (Figure 3, Supplemental Table 1). The most frequent reason for upgrading a VUS (32.6% [29/89]) to likely pathogenic or pathogenic was applying de novo criteria (pathogenic strong 2 [PS2]; de novo with both maternity and paternity confirmed). Parental testing within Simons Searchlight aided in an upgrade reclassification for 2 of these probands, and parental testing was completed for the other 27 probands in their medical clinic. Missense variants submitted 3 or more times for unique probands were considered recurrent within our database, accounting for 18.0% of revisions (16/89). Recurrence of the variant in the literature or ClinVar (case series) was applied to upgrade 6.7% (6/89) of upgraded variants. The scientific upgrade of a candidate gene to be disease associated allowed for an upgrade of 20.2% (18/89), as well as the identification of mutational hotspots reclassified 3.4% (3/89) VUS. Published functional data drove the reclassification of 3.4% (3/89) of cases, and clinical RNA sequencing for 2.2% (2/89). Updated in silico functional predictions allowed for the revision of 3.4% of missense variants (3/89). Application of additional ACMG criteria drove the remaining 7.9% (7/89) of upgrades. The additional application of moderate evidence of pathogenicity was applied for 4 cases that were assumed de novo (pathogenic moderate 6 [PM6]; assumed de novo because of other de novo cases within the database or literature), or a combination of moderate and supporting evidence of pathogenicity for the other that includes absent from population controls, missense variant in a gene with low frequency of benign missense variation, and multiple lines of computational evidence supporting a deleterious effect or patient’s phenotype (pathogenic moderate 2 [PM2]; absent from large population studies, and/or pathogenic supporting 2 [PP2]; typically missense, and/or pathogenic supporting 3 [PP3]; predicted to be damaging, and/or pathogenic supporting 4 [PP4]; evidence of family history) that aided in classification. Finally, of the variants that were downgraded to benign, parental testing within Simons Searchlight resulted in 6.6% (1/15) being downgraded.

Cascade genetic testing provided reclassification in 18.6% of families with a monogenic likely pathogenic or VUS variant (13/70); of those revised variants, 92.3% were upgraded (12/13, Supplemental Figure 1). In our cascade genetic testing process, we provided parental testing for 17 probands. Within the trios, 2 VUS were upgraded to likely pathogenic, 10 likely pathogenic variants were upgraded to pathogenic, and 5 variants were not reclassified. Variants that were not reclassified were found to be inherited from a parent.

**Figure 4:**
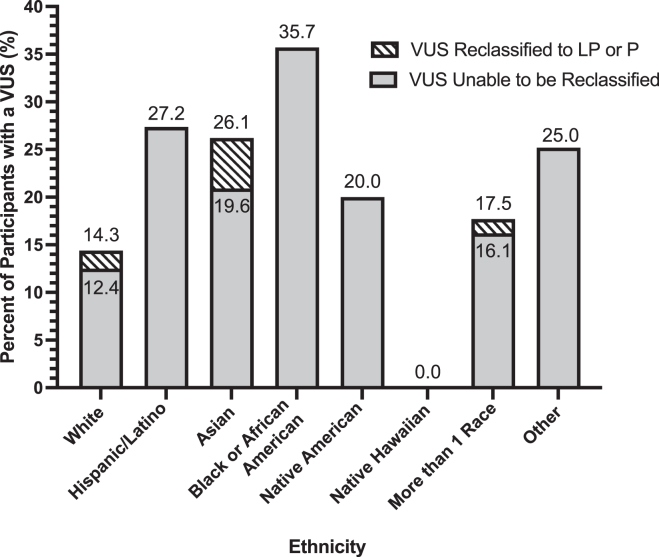
**Frequency of participant ethnicity at the time a VUS is submitted and how often VUS was reclassified after review (shaded).** LP, likely pathogenic; P, pathogenic; VUS, variants of uncertain significance.

Because non-Europeans are more likely to receive a VUS in their genetic testing because of the bias of European individuals in reference databases,^18^^,^^19^ we investigated self-reported race/ethnicity and reclassification. Categories of race and ethnicity were collected according to the USA census categories of White, Hispanic or Latino, Asian, Black or African American, Native American/Alaska Native, Native Hawaiian or Other Pacific Islander, more than 1 race, and Other. There were 33 variants submitted as pathogenic or likely pathogenic that were downgraded to a VUS and were included in the postrevision numbers. Participants who selected Other indicated their child was adopted or did not indicate any details further. White participants were less likely to have a VUS variant at the time of registration 14.3% (132/921) when compared with all other races/ethnicities 23.3% (77/331) (χ^2^ [1, *N* = 1461] = 9.63, *P* < .01) with a χ^2^ test (Figure 4). Participants historically underrepresented in the reference genome, Hispanic, Black or African American, and Native American/Alaska Native race/ethnicities more frequently had variants that remained VUS (Figure 4).

**Figure 5:**
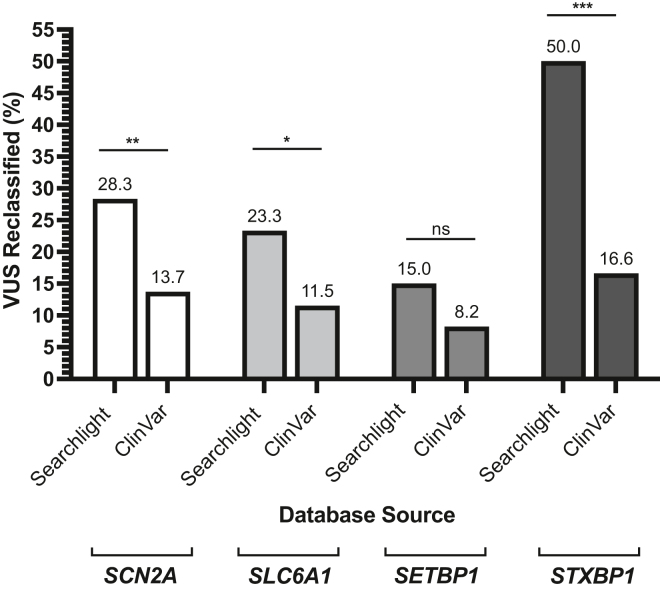
**Percent VUS reclassified within Simons Searchlight (Searchlight) compared with ClinVar.** ∗*P* < .05, ∗∗*P* < .01, and ∗∗∗*P* < .001, using a χ^2^ test. VUS, variants of uncertain significance.

We questioned how often genetic variant reclassification is occurring clinically for our top monogenic conditions with a VUS submitted compared with variant resubmissions in the ClinVar database. Therefore, we reviewed our top 4 monogenic conditions with VUS variants submitted to ClinVar as of December 2022 to quantify how many variants had other classifications within ClinVar compared with Simons Searchlight as of December 2022. The frequency of VUS reclassification for *SCN2A*, *SLC6A1*, *SETBP1*, and *STXBP1* within Simon Searchlight was higher for all these conditions (Figure 5). Using a χ^2^ test, we found that participants in Simons Searchlight had more VUS reclassified compared with ClinVar if they had a variant in *SCN2A* (χ^2^ [1, *N* = 879] = 7.49, *P* < .01), *SLC6A1* (χ^2^ [1, *N* = 265] = 4.86, *P* < .05), or *STXBP1* (χ^2^ [1, *N* = 217] = 15.78, *P* < .001). Total revisions included VUS reclassification to likely benign, benign, likely pathogenic, or pathogenic for both databases. The frequency of VUS reclassifications in Simons Searchlight for these genetic conditions was higher than the frequency that has been analyzed for the full ClinVar data set as of January 2023 (6%) (Ruscheinski A, Reimler AL, Ewald R, Uhrmacher AM. Towards analyzing the reclassification dynamics of ClinVar variants. *bioRxiv*. 2023. https://doi.org/10.1101/2023.01.24.525342). This suggests that reclassifications submitted by ClinVar users might not be as frequent for variant interpretation compared with review by disease experts compiling variants on a global scale.

## Discussion

A concern in the medical community is that increased genetic testing will result in a greater burden of uncertainty with more VUS. The inability to reclassify VUS is often due to rare variants and missense variants for which functional effects can be difficult to predict and functional data are sparse. Our study systematically verifies genetic test reports to ensure the appropriateness of participant inclusion based upon genetic diagnosis. We uniquely focus on neurodevelopmental disorders, allowing for the analysis of variant interpretation and reclassification for this clinical indication. CNVs were less likely to be reclassified because of the study’s inclusion of recurrent CNVs with well-defined phenotypes. However, variant classification for monogenetic neurodevelopmental conditions is more complex, with a dramatic increase in the number of conditions over the past 10 years.^20^ Variants in monogenic conditions were more likely to be revised (18.7%) compared with recurrent CNVs (1.7%), and only 4.3% of VUS were downgraded. Variant classification across neurodevelopmental conditions is expected to improve over time with more reference data and more data from disease cohorts.

VUS in *SCN2A*, *SLC6A1*, and *STXBP1* in Simons Searchlight were more likely to be reclassified compared with variants in the same genes in ClinVar. The increased frequency of variant reclassification in our analysis was driven largely by de novo criteria (PS2) either by providing testing through Simons Searchlight or receiving this information from the participant’s medical provider. Additionally, we have aggregated rare variants for neurodevelopmental disorders from the literature. Of the VUS upgraded to likely pathogenic or pathogenic, 18.0% were driven by recurrence for the missense variant. The majority of VUS not reclassified in our system are of unknown inheritance and unique missense variants. There are many reasons why parents do not complete testing even when we provide the free service including: that parents assume the VUS classification is a clinical diagnosis; parents have concerns that they may have passed the variant to their child, which provokes feelings of guilt or blame; lack of availability of a biological parent; or location outside of the United States and difficult specimen shipment. Investigating inheritance to aid in variant classification is not a novel concept; however, 18.6% of monogenic families who had VUS or likely pathogenic results from targeted testing could have been reclassified if parents had been offered testing in a clinical setting. Simons Searchlight contributes variant classification to ClinVar for monogenetic conditions to facilitate collaboration and genetic interpretation accuracy for clinics and laboratories worldwide.

The higher frequency of finding a VUS when performing genetic testing in non-Europeans compared with European participants leads to a higher frequency of VUS in non-Europeans broadly.^18^ Although only 36.8% of submitted VUS were for non-White participants (77/209), our data suggest that there is greater difficulty with reclassifying VUS for these participants. Studies have identified a bias in the reference genomes available to the genetic community, limiting the capacity for clinicians to support patients equitably.^21^ Within our data, the high rate of reclassification of self-reported Asian participants was due to a few reclassifications of a small number of participants. There were 12 VUS of 46 Asian participants total (26.1%) and 3 were reclassified; however, this sample size is modest. As we continue to collect self-reported race and ethnicity information, and our study expands, we will reassess the evolution of variant interpretation across the neurodevelopmental community.

Simons Searchlight’s independent variant reclassification demonstrates the utility and importance of regular variant review for neurodevelopmental genetic conditions. Increasing knowledge about gene function and mechanism of disease, expanding case series, and availability of segregation data all provide data for reclassifications. This analysis also suggests that such reclassifications are not occurring within clinical care and are likely to be an unmet need as genetic testing continues to expand.^22^

### Limitations

Simons Searchlight has reviewed and tracked variant information over time. VUS downgrades to benign or likely benign before 2019 were not captured within our database; therefore, there may be even more VUS reclassified. Finally, race and ethnicity data collected according to US census guidelines may not be relevant to all participants living in other countries because descriptions and definitions differ by country.

## Data Availability

Deidentified data will be made available to qualified researchers who submit and provide a valid research question. Please direct inquiries to https://base.sfari.org.

## Conflict of Interest

The authors declare no conflicts of interest.

## Supplementary Material

Supplemental Figure 1 If inheritance was not known, cascade testing was offered to participants to aid in the reclassification of variants.

Supplemental Table 1 Genetic laboratory reports were submitted from 71 different countries (tab 1) for 91 genetic conditions (tab 2). Criteria applied for revisions in classification are itemized for each variant (tab 3).
