# Supplementary figures and images for: Improvement of variant reclassification in genetic neurodevelopmental conditions
Source: Genet Med Open. 2024 Apr 9;2:101845. doi: 10.1016/j.gimo.2024.101845 (PMC11613566; doi:10.1016/j.gimo.2024.101845)

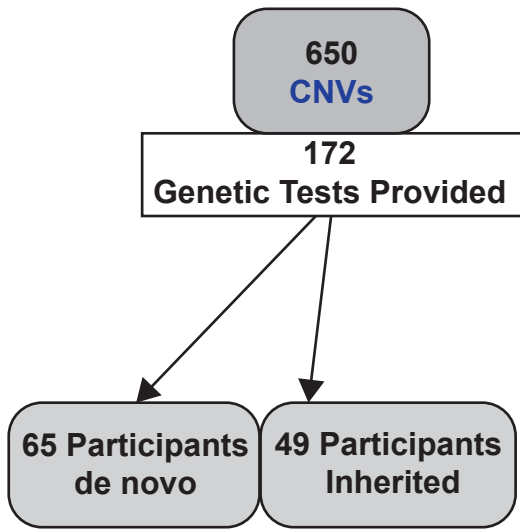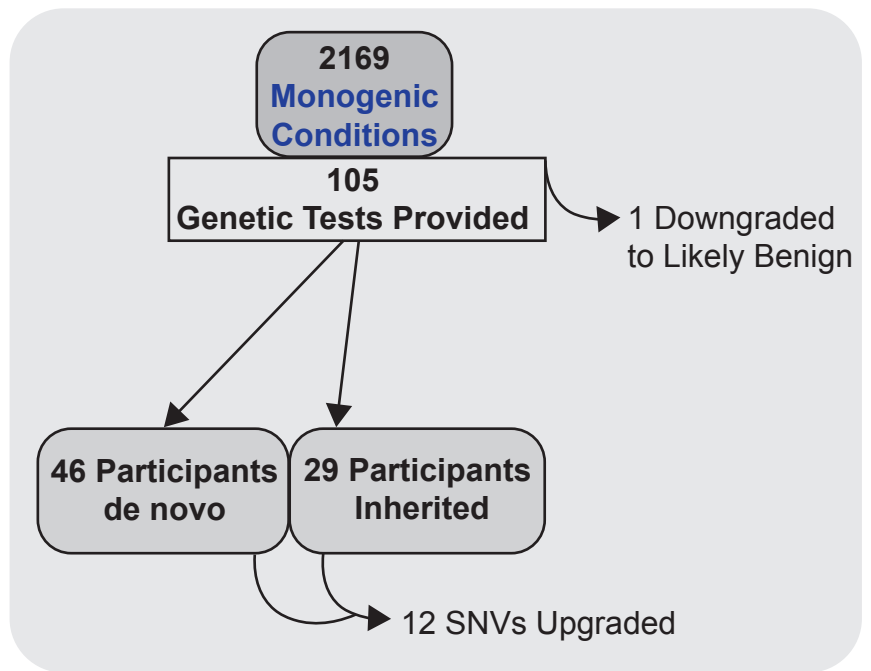

Supplement: Supplemental Figure 1 — If inheritance was not known, cascade testing was offered to participants to aid in the reclassification of variants. [file mmc1.pdf]
